# The effect of intracerebroventricular injection of histamine in visceral nociception induced by acetic acid in rats

**DOI:** 10.4103/0253-7613.70157

**Published:** 2010-10

**Authors:** Ali Zanboori, Esmaeal Tamaddonfard, Ali Mojtahedin

**Affiliations:** Department of Basic Sciences, Division of Physiology, Faculty of Veterinary Medicine, Urmia University, Urmia, Iran

**Keywords:** Brain, chlorpheniramine, histamine, ranitidine, rats, visceral nociception

## Abstract

**Objective:**

This study was designed to investigate the role of brain histamine and H1 and H2 receptors in mediating the central perception of visceral pain in rats.

**Materials and Methods:**

In conscious rats implanted with a lateral brain ventricle cannula, the effect of intracerebroventricular (i.c.v.) injection of histamine (2.5, 10, and 40 μg), and chlorpheniramine and ranitidine at the same doses of 5, 20, and 80 μg were investigated on visceral pain. Visceral nociception induced by intraperitoneal (i.p.) injection of acetic acid (1 mL, 1%), and the number of complete abdominal wall muscle contractions accompanied with stretching of hind limbs (writhes) were counted for 1 h.

**Results:**

Histamine at doses of 10 and 40 μg and chlorpheniramine and ranitidine at the same doses of 20 and 80 μg, significantly decreased the numbers of writhes (*P* < 0.05). Pretreatment with chlorpheniramine and ranitidine at the same dose of 80 μg, significantly prevented histamine (40 μg)-induced antinociception (*P* < 0.05).

**Conclusion:**

The results of this study suggest that brain histamine may be involved in modulation of visceral antinociception through both central H_1_and H_2_receptors.

## Introduction

Pain arising from distension, ischemia, and inflammation of the viscera, such as stomach, kidney, gallbladder, urinary bladder, and intestines, constitute a large part of clinically treated pains.[1] Vagal afferent system and spinal ascending pathways including spinothalamic tract and dorsal column system convey visceral nociceptive information to the higher centers of the central nervous system.[2,3] Many of brain nuclei and regions such as nucleus gracilis, ventroposterolateral nucleus of thalamus, locus coeruleus/subcoeruleus (LC/SC), anterior and posterior cingulated cortex, and somatosensory cortex participate in the central perception of visceral pain.[4–6] Brain chemical messengers including serotonin, noradrenaline, dopamine, opiates, cytokines, and glutamate are involved in the brain modulation of visceral nociception.[4–6]

Several lines of evidence suggest that brain histamine may be involved in the central perception of pain. Intracerebroventricular (i.c.v.) injection of histamine has been shown to produce antinociception in a hot plate and paw pressure,[7] formalin,[8,9] neuropathic,[10] and trigeminal[11] pain tests in mice and rats. Intrahippocampal microinjection of histamine produced an antinociceptive effect in the formalin-induced orofacial pain in rats.[12] It is recognized that the action of brain histamine on pain modulation is mediated through histamine H_1_, H_2_, and H_3_ receptors.[7–12]

There are no reports regarding the direct effect of brain histamine and the involvement of its central H_1_and H_2_receptors in the acetic acid-induced visceral nociception in rats. In one study, centrally administered histamine produced antinociception in the acetic acid-induced writhing test in mice.[7] Moreover, the involvement of brain histamine H_1_and H_2_receptors was reported in the phenylquinone- and acetic acid-induced visceral nociceptive tests in mice.[13,14] Therefore, this study was designed to investigate the effect of i.c.v. injection of histamine, chlorpheniramine (a histamine H_1_-receptor antagonist), and ranitidine (a histamine H_2_-receptor antagonist) on the acute visceral nociception induced by i.p. injection of acetic acid in rats.

## Materials and Methods

### Animals

Healthy adult male Wistar rats, weighing 200–220 g were used in this study. Rats were maintained in polyethylene cages with food and water available *ad libitum*, in a laboratory with controlled ambient temperature (23 ± 0.5°C) and under a 12 h light–dark cycle (lights on from 07:00 h). Experiments were carried out between 9:00 and 13:00 h. All experimental procedures were approved by the Veterinary Ethics Committee of the Faculty of Veterinary Medicine of Urmia University and were performed in accordance with the National Institutes of Health Guide for Care and Use of Laboratory Animals. Fortytwo rats were divided into seven groups with six rats in each group. The following treatments were administered: (i) normal saline; (ii) histamine (2.5 and 10 μg); (iii) histamine (40 μg); (iv) chlorpheniramine (5, 20, and 80 μg); (v) ranitidine (5, 20, and 80 μg); (vi) chlorpheniramine (80 μg) plus histamine (40 μg); and (vii) ranitidine (80 μg) plus histamine (40 μg). Therefore, each rat received 1, 2, or 3 different doses in one drug treatment and the gap between drug treatments was 5 days.

### Drugs and Chemicals

Drugs and chemicals used in this study included histamine dihydrochloride (Merck, Darmstadt, Germany), chlorpheniramine maleate (Sigma-Aldrich Co., Steinheim, Germany), ranitidine hydrochloride (Sigma-Aldrich Co., Steinheim, Germany), and formaldehyde solution (37%, Merck, Darmstadt, Germany). The drugs were dissolved in normal saline 1 h before i.c.v. injections.

### Surgery

After a 15-day adaptation period, each rat was anaesthetized with a mixture of ketamine (80 mg/kg) and xylazine (10 mg/kg) injected i.p., and then placed in a stereotaxic apparatus (Stoelting, Wood Lane, IL, USA). The scalp was incised, and the skull was levelled off around the bregma. A 22 gauge, 12 mm stainless-steel guide cannula was inserted in the right lateral ventricle of the brain. The tip of the cannula was aimed at the following coordinates: 0.8 mm posterior to the bregma, 2 mm lateral to the midline, and 4 mm below the top of the skull.[15] The cannula was then fixed to the skull using three screw and dental acrylic. A 12.5-mm stylet was inserted in the cannula to keep it patent prior to injection. Animals were allowed a 10-day recovery period before experiments were initiated.

### Drug Injection

For i.c.v. injections of normal saline (control), histamine, chlorpheniramine, and ranitidine, a 28 gauge, 12.5 mm injection needle was attached to a 30-cm polyethylene tube fitted to a 5-μL Hamilton syringe. Then, the rat was restrained by hand, the stylet was withdrawn, and the injection needle was inserted into the guide cannula. The volume of the solutions to be injected into lateral ventricle was 1 μL, and the injection was made over a period of 60 s.

### Visceral Nociception

The induction of visceral nociception was performed using writhing test. For this purpose, each rat was placed inside a plexiglass chamber (40 × 30 × 20 cm^3^) for an acclimatization period of 30 min. At the end of this period, 1 mL of 1% acetic acid was i.p. injected using a 25-gauge injection needle, and the numbers of writhes were counted for 1 h. A writhe was defined as a wave of the contraction of the abdominal wall muscles followed by extension of the hind limbs.[16,17] In control rats, the i.p. injection of appropriate amount of normal saline was performed.

### Cannula Verification

For confirmation of the placement of the cannula in the lateral ventricle of the brain, at the end of experiments, the rats were i.c.v. injected with 10 μL methylene blue and then were deeply anesthetized with the high dose of ether and decapitated. The brains were removed and placed in formaldehyde (10%) solution. After 24 h, the brains were sliced into 1 mm slices and the place of the tip of the cannula and distribution of the dye in the lateral ventricle were visually controlled. Data from rats with an incorrect placement of the cannula were excluded from the data analysis.

### Statistical Analysis

All the values are expressed as the mean ± SEM. The data were analyzed by using one-way analysis of variance (ANOVA) followed by Duncan’s test. Statistical significance was considered at *P* < 0.05.

## Results

**Figure 1:**
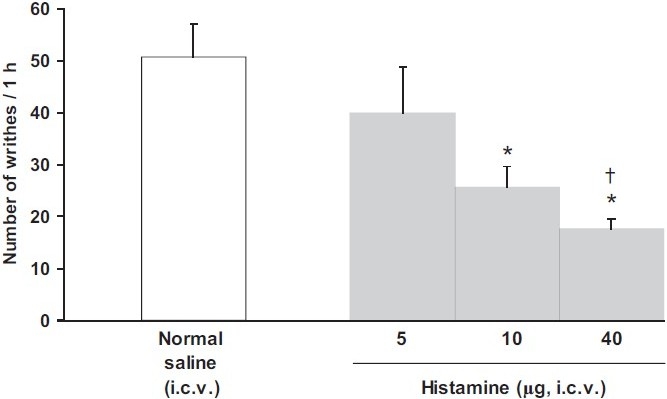
Effect of i.c.v. injection of histamine on the numbers of writhes induced by acetic acid in rats. Each column represents mean ± SEM (n = 6 rats for normal saline, six rats for histamine 2.5 and 10 μg, and six rats for histamine 40 μg), *P < 0.05 vs. normal saline and histamine (2.5 μg), †*P* < 0.05 vs. histamine at the dose of 10 μg (one-way ANOVA followed by Duncan’s test), i.c.v.: intracerebroventricular.

**Figure 2:**
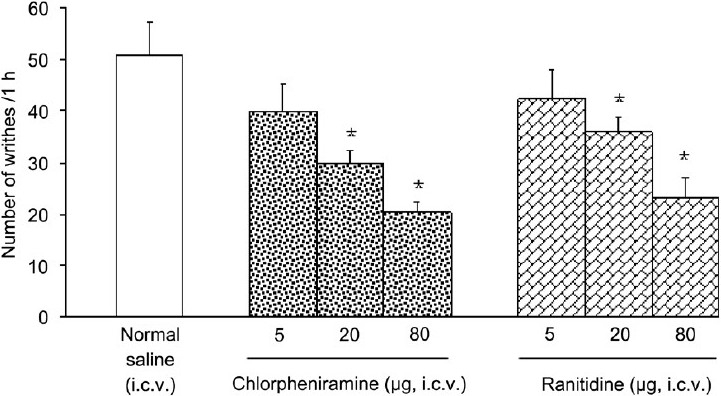
Effect of i.c.v. injection of chlorpheniramine on the numbers of writhes induced by acetic acid in rats. Each column represents mean ± SEM (n = 6 rats for normal saline, six rats for chlorpheniramine and six rats for ranitidine). *P < 0.05 vs. normal saline (one-way ANOVA followed by Duncan’s test), i.c.v.: intracerebroventricular.

I.c.v. injection of histamine at doses of 10 and 40 μg, but not at a dose of 2.5 μg, significantly decreased the numbers of writhes induced by acetic acid. A significant difference was observed between the effects of histamine used at doses of 10 and 40 μg (F_(3,20)_= 6.390, *P* < 0.05, one-way ANOVA)[Figure 1]. I.c.v. injection of chlorpheniramine at doses of 20 and 80 μg, but not at a dose of 5 μg significantly reduced the number of writhes (F_(3,20)_= 8.554, *P* < 0.05, one-way ANOVA). Similar results were obtained from i.c.v. injection of ranitidine at doses of 5, 20, and 80 μg (F_(3,20)_= 5.721, *P* < 0.05, one-way ANOVA)[Figure 2].

**Figure 3:**
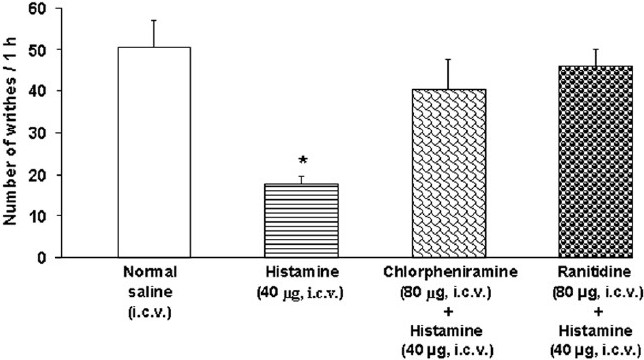
Effect of i.c.v. injection of ranitidine on the numbers of writhes induced by acetic acid in rats. Each column represents mean ± SEM (n = 6 rats for normal saline, six rats for histamine, six rats for chlorpheniramine plus histamine, and six rats for ranitidine plus histamine). *P < 0.05 vs. other groups (one-way ANOVA followed by Duncan’s test), i.c.v.: intracerebroventricular.

I.c.v. pretreatments with chlorpheniramine and ranitdine at the same dose of 80 μg significantly inhibited the histamine (40 μg)-induced antinociception (F_(3,20)_= 7.737, *P* < 0.05, one-way ANOVA)[Figure 3].

## Discussion

In this study, i.c.v. injection of histamine produced antinociception in the acetic acid-induced visceral nociception in rats. The cell bodies of histaminergic neuronal system are found only in the tuberomammillary nucleus (TMN) of the hypothalamus, and their fibers and terminals innervate the entire central nervous system.[18] The areas such as the external layers of the dorsal horn of the spinal cord, the preaquductal gray and raphe nucleus, known to be involved in the nociceptive control,[19] are also innervated by the histaminergic system of the hypothalamus.[18] Evidences taken from various acute and chronic pain tests, such as hot plate, formalin, neuropathic, and trigeminal pain tests suggest that the brain histamine influences the central perception of pain.[7–11] On the central effect of histamine on visceral pain, it was reported that i.c.v. injection of histamine produced antinociception in the abdominal constriction test in mice.[7] Moreover, i.c.v. injection of SKF 91488 (a histamine-N-methyltransferase inhibitor) suppressed nociception induced by intraperitoneal (i.p.) injection of acetic acid in mice.[20]

In this study, both histamine H_1_and H_2_receptor blockers, chlorpheniramine and ranitidine, produced antinociception in the absence of histamine, but in the presence of histamine, prevented the histamine-induced antinociception. This indicates that both H_1_ and H_2_antagonists may have analgesic properties. Histamine H_1_ and H_2_ presynaptic and H_3_ postsynaptic receptors are distributed, approximately, in the all regions of the central nervous system and are involved in the histamine actions in the central nervous system.[18] Both histamine H_1_ and H_2_ receptors may involve in the brain histamine-induced antinociception since mutant mice lacking the histamine H_1_ and H_2_receptors, showed fewer nociceptive responses in various pain tests.[21,22] It has been reported that i.c.v. injection of 2-(3-triflouromethylphenyl) histamine dihydrogenmaleate, 2-thiazolylethylamine (H_1_-receptor agonists), and pyrilamine (H_1_-receptor antagonist) produce hypernociception and antinociception, respectively, which suggests that H_1_receptor activation increases sensitivity to noxious stimuli.[23] In addition, the tricyclic compound, ReN 1869, a novel histamine H_1_receptor antagonist that penetrates the blood–brain barrier, has been found to induce antinociception in chemical (formalin, capsaicin, and phenylquinone writhing) but not thermal (hot plate and tail flick) tests of nociception.[13] In the hot plate test in rats, i.c.v. injections of H_2_agonist (4-methylhistamine) and antagonists (cimetidine and ranitidine) enhanced the pain threshold.[14] In another study, it was found that intracerebral microinjection of temelastine (H_1_-receptor antagonist) and cimetidine into the preaquductal gray or into the raphe nucleus prevented the histamine-induced antinociception.[24] In addition, subcutaneous (s.c.) injections of mepyramine (H_1_-receptor antagonist) and zolantidine (H_2_-receptor antagonist) that cross the blood–brain barrier produced antinociception in the acetic acid-induced writhing test in mice.[25] Intrahippocampal microinjection pretreatments with mepyramine and ranitidine blocked the antinociceptive effect induced by intrahippocampal microinjection of histamine in the orofacial pain region in rats.[12] In the formalin test in rats, i.c.v. injection of histamine produced antinociception and pretreatments with mepyramine and famotidine blocked the histamine-induced antinociception.[9]

The antinociception induced by i.c.v. injection of the high doses of chlorpheniramine (20 and 80 μg), observed in this study may be related to its side effects, because chlorpheniramine belongs to the first class of H_1_ antihistamines, and sedation, drowsiness, and poor motor coordination are the side effects of first class antihistamines.[26] In this study, ranitidine produced analgesia in the absence of histamine. In the hot plate test in rats, the pain threshold enhancement was reported after i.c.v. injections of histamine H_2_ receptor agonists (4-methylhistamine and dimaprit) and antagonist (cimetidine). They suggested that antinociceptive activity of cimetidine was not in relation to the specific blockade of H_2_ receptors.[14] However, in the central action of cimetidine on pain perception, possible involvement of other mechanisms such as serotonergic, muscarinic, nicotinic, dopaminergic, gabaergic, and adrenergic as well as histaminergic need to be considered.[27,28]

In conclusion, the present data show that besides the analgesic activity, chlorpheniramine, and ranitidine prevented the histamine-induced antinociception, i.e., brain histamine through its central H_1_ and H_2_ receptors is able to produce visceral antinociception in rats.
